# Visible-Light-Controlled Thermal Energy Storage and Release: A *Tetra*-*Ortho*-Fluorinated Azobenzene-Doped Composite Phase Change Material

**DOI:** 10.3390/molecules30173576

**Published:** 2025-08-31

**Authors:** Yating Zhang, Jing Qi, Jun Xia, Fei Zhai, Liqi Dong

**Affiliations:** 1 Key Laboratory of Pollution Exposure and Health Intervention of Zhejiang Province, Interdisciplinary Research Academy, Zhejiang Shuren University, Hangzhou 310021, China; 2 College of Environment, Zhejiang University of Technology, 18 Chaowang RD, Hangzhou 310014, China; 3 Faculty of Social Sciences and Liberal Arts, UCSI University, Kuala Lumpur 56000, Malaysia; 4 Shandong Laboratory of Advanced Materials and Green Manufacturing at Yantai, Yantai Zhongke Research Institute of Advanced Materials and Green Chemical Engineering, Yantai 264006, China; 5 Zhejiang Collaborative Innovation Center for Full-Process Monitoring and Green Governance of Emerging Contaminants, Hangzhou 310021, China

**Keywords:** phase change materials, azobenzene, photoinduced supercooling, visible-light-controlled energy storage, optically-triggered heat release

## Abstract

Organic phase change materials (OPCMs) offer high energy density for thermal storage but suffer from crystallization kinetics dependent on ambient temperature, leading to uncontrolled heat release and limited storage lifetime. Although doping OPCMs with azobenzene (Azo) derivatives enables optically controlled energy storage and release, existing systems require UV irradiation for *E*-to-*Z* isomerization. This UV dependency seriously hinders their development in practical solar applications. Herein, we develop a visible-light-responsive Azo@OPCM composite by doping *tetra*-*ortho*-fluorinated azobenzene into eicosane. Systematic characterization of composites with different dopant ratios via UV–visible spectroscopy and differential scanning calorimetry reveals that green-light irradiation drives *E*-to-*Z* isomerization, achieving 97–99% *Z*-isomer conversion. This photoisomerization could introduce supercooling through photo-responsive energy barriers generated by *Z*-isomer, allowing thermal energy storage at lower temperatures. Subsequent blue-light irradiation triggers *Z*-to-*E* reversion to eliminate supercooling and enable optically controlled heat release. Additionally, by regulating the molar ratios of dopant, the optimized composites achieved 280.76 J/g energy density at 20% molar doping ratio, which surpassed that of pure eicosane and the reported Azo-based photothermal energy storage system. This work establishes a complete visible-light-controlled energy harvesting–storage–release cycle with significant potential for near-room-temperature solar thermal storage applications.

## 1. Introduction

Latent heat storage based on organic solid–liquid phase change materials (OPCMs) represents one of the most efficient energy storage technologies, offering high energy density, favorable physicochemical properties, and abundant natural precursors [1,2]. These features make OPCMs highly promising for diverse applications, including solar thermal utilization [3,4,5], building temperature regulation [6,7,8], smart windows [9,10], intelligent textiles [11,12], and thermal management of electronic devices [13,14]. However, latent heat storage in OPCMs is fundamentally limited by their exclusive dependence on ambient temperature for crystallization kinetics, resulting in uncontrolled heat release. Once the latent heat is stored in the liquid phase, the system temperature must be continuously maintained above the crystallization point to prolong thermal storage duration [15]. Without continuous energy input or adequate thermal insulation, spontaneous crystallization will inevitably occur, causing rapid latent heat dissipation to ambient and significantly curtailed storage duration. Consequently, conventional OPCMs face several critical challenges when employed in practical applications, such as rapid heat loss, narrow liquid-phase stability windows, limited storage durations, and uncontrolled heat release [16,17,18]. These limitations fundamentally impede long-term photothermal energy storage, long-distance thermal transport, and on-demand heat release, especially in environments characterized by short daylight periods or significant diurnal temperature fluctuations. Therefore, developing novel phase-transition control strategies that decouple crystallization from ambient temperature is imperative to enable stable photothermal storage and optically triggered, controllable heat release.

Recently, a promising strategy for achieving long-term thermal storage and controllable heat release has garnered significant attention: doping OPCMs with photo-liquefiable azobenzene(Azo) derivatives capable of reversible photoinduced crystal-to-liquid transitions (PCLT) [15,19,20]. These molecular photo-switches undergo reversible *E*-to-*Z* isomerization upon UV irradiation, accompanied by a concomitant crystal-to-liquid phase change and substantial alterations in optical and physical properties [21,22,23,24,25,26]. This photoisomerization imposes a tunable energy barrier that prevents the spontaneous crystallization of the host OPCM, enabling the composite phase change material (CPCM) to maintain its liquid phase at temperatures below the intrinsic crystallization point of the pristine material. Consequently, thermal energy stored within the supercooled liquid phase can be preserved for extended durations at lower temperatures. Crucially, this metastable state can be overcome by visible-light illumination, which triggers *Z*-to-*E* isomerization, reverts the dopant to its crystalline *E*-configuration, and initiates crystallization of the CPCM, thereby releasing the stored latent heat [27,28,29] The pioneering work of Han et al. demonstrated that UV-irradiated azobenzene dopants in tridecanoic acid preserved the liquid phase significantly below the original crystallization point through enhanced molecular interactions [15]. This photo-switch/OPCM hybrid system exhibited exceptional cycling stability (over 100 cycles) and extended thermal storage duration (over 10 h under supercooling), whereas the pristine OPCMs crystallized within minutes when cooled below their crystallization temperature. Subsequent work by Han’s research group expanded this strategy to diverse aliphatic OPCMs [30]. UV activation of the Azo dopants induced supercooling of the CPCMs, facilitating extended thermal storage at lower temperatures. By comparing phase change behaviors across diverse materials, the group delved into the mechanism of this process and the design principles for such hybrid systems. Concurrently, Feng’s research group developed alkyl-grafted Azo/tetradecane composites enabling optically triggered synchronous release of phase change enthalpy and photothermal energy at remarkably low temperatures (−1.96 to −6.71 °C), achieving a high energy density of 207.5 J/g. To validate practical utility in distributed energy systems, an annular device was fabricated for energy utilization, including light absorption, low-temperature storage, and optically triggered heat release [28,31]. Feng’s group further designed a stable two-phase hybrid system by combining the meta-azopyridine polymer with OPCMs, leveraging hydrogen bonds and van der Waals interactions to collectively harness phase change energy and photothermal energy [32]. The OPCMs not only contribute additional phase change latent heat but also serve as a solvent, providing sufficient free volume for the photo-induced isomerization of the azopyridine chromophores, which successfully circumvents the low charging efficiency in the condensed state and reliance on solvent-assisted charging in traditional Azo-based photothermal energy storage. Jiang et al. successfully synthesized high-performance CPCMs by incorporating photo-liquefiable Azo into tetradecyl alcohol or lauric acid. These composite materials exhibit high energy density (239 J/g), a moderate degree of supercooling (4–12 °C), stable thermal storage (10 h), and blue-light-controlled heat release [33,34].

However, a critical limitation persists in current Azo@OPCM composite systems: the formation of the energy barrier necessary to stabilize the liquid phase for thermal energy storage typically requires high-intensity ultraviolet (UV) irradiation to drive the *E*-to-*Z* isomerization. This requirement presents a significant obstacle to practical operation under natural sunlight conditions. Consequently, the development of Azo@OPCM composite capable of visible-light-induced *E*-to-*Z* isomerization has emerged as a research direction aligned with real-world needs. Fortunately, recent studies by our group [35,36] and others [37,38,39,40,41] have highlighted the *tetra*-*ortho*-functionalization of Azo moieties with halogen groups. This strategy successfully achieves a red-shift of the n–π* transition in the *E* isomers through intramolecular interactions between the *ortho*-substituents and N=N band, enabling *E*-to-*Z* isomerization through visible light irradiation [42]. Although the synthesis and characterization of such visible-light-responsive Azo photo-switches have been extensively investigated, previous efforts [36,43] have primarily focused on molecular-level properties of a single photothermal molecule, demonstrating its ability to efficiently store visible-light energy and release heat upon light stimulation, achieving relatively high energy storage density, long storage lifetime, and remarkable cycling stability. Nevertheless, their integration as dopants into OPCM matrices to exploit their exceptional optical properties for actively regulating phase transition kinetics remains largely unexplored.

Herein, we design and fabricate a visible-light-responsive Azo@OPCM composite by doping *tetra*-*ortho*-fluorinated azobenzene (4FAzo) into eicosane. We establish a complete energy harvesting–storage–release pathway for this system. Systematic UV-Vis spectroscopy investigations confirm that green light (530 nm) and blue light (430 nm) irradiation drive reversible *E*-to-*Z* isomerization and *Z*-to-*E* reversion in the 4FAzo dopant, respectively. DSC analysis reveals that *Z*-rich 4FAzo introduces photo-responsive energy barriers within eicosane, inducing supercooling for lower temperature thermal storage. Subsequent blue light irradiation eliminates supercooling through *Z*-to-*E* reversion, triggering optically controlled heat release. The regulating effects of variational molecular configurations and molar ratios on the energy density and photoinduced supercooling were further surveyed and discussed. Furthermore, by regulating 4FAzo molar ratios in accordance with crystallization and isomerization dynamics, we achieve tunable supercooling, phase-change enthalpy, and photothermal energy storage, ultimately attaining a maximum energy density of 280.76 J/g (20% molar doping ratio), surpassing that of pure eicosane. This work creates new opportunities for near-room temperature solar thermal storage applications.

## 2. Results and Discussion

### 2.1. Molecule Design and Energy Havesting–Storage–Release Mechanism

**Figure 1:**
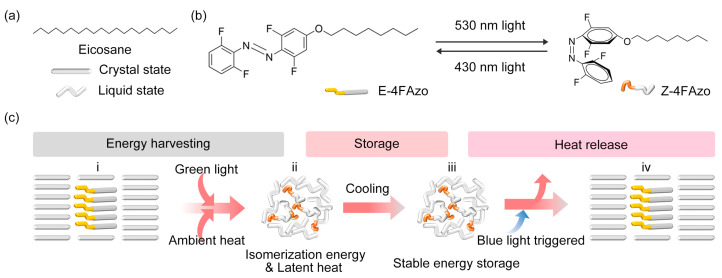
Chemical structure of eicosane PCM (**a**) and the 4FAzo dopant in each isomeric form (**b**). (**c**) Schematic illustration of optically controlled energy storage and release cycle in the composite of eicosane and 4FAzo photo-switching dopants.

In this study, we chose commercial eicosane as the OPCM component in OPCM@Azo composite due to its optimal phase transition properties (melting point ~36–40 °C), high latent heat capacity (up to 247 J/g theoretically), and structural compatibility in composite forms (Figure 1a) [44,45,46]. In the meantime, the selection of Azo dopant has been seriously considered as well. On the one hand, in recent years, it has been demonstrated that *tetra*-*ortho*-fluorinated Azo exhibits the ability of effectively green-light-induced *E*-to-*Z* isomerization [35,37,40,47,48,49]. Such a phenomenon endows the compound with the notable capability to capture and store energy from both green regions of the visible light spectrum. Additionally, according to previous research [35,47], The *Z*-isomer of Azo with *tetra*-*ortho*-fluorinated structure present a significant half-life of beyond 700 days due to the thermal stability of the *Z* configuration, which gives us an expectation for long-term energy storage in CPCM using such Azo as a dopant. Therefore, the *tetra*-*ortho*-fluorinated Azo was chosen as the photo-switching structure. On the other hand, the character of reversible photoinduced *E*-crystal to *Z*-liquid transitions for a photo-liquefiable Azo molecule was usually obtained by decorating an alkyl chain on the *para*-position of the Azo molecule [36,50,51,52,53,54]. The melting point of the *E*/*Z* mixture decreases due to the *Z*-liquid, which facilitates more *E*-crystal convert to *Z*-liquid [35,55]. Thus, the integration of fluorine at the *ortho*-position and alkoxy chain at the *para*–position could yield a visible-light-PCLT Azo, which presents a promising candidate for visible-light photon energy and ambient heat co-harvesting, long-term energy storage, and high-temperature heat release OPCM@Azo composite (Figure 1b).

The optically controlled energy storage and release cycle initiates with the co-harvesting of thermal and photonic energy by the solid eicosane@4FAzo composite (Figure 1c(i)). Under green light irradiation, photothermally generated heat raises the eicosane temperature above its melting point, inducing a solid-to-liquid phase transition. Concurrently, the 4FAzo dopant undergoes photoinduced *E*-to-*Z* isomerization upon absorbing green-light photons. Given the distinct melting points of the isomers (typically, the *Z*-isomer exhibits a significantly lower melting point than the *E*-isomer), this isomerization is accompanied by a crystalline-to-liquid phase transition of dopant. Critically, this phase transition requires spontaneous absorption of ambient heat to satisfy energy conservation. Consequently, the composite transforms into a mixture of molten eicosane and liquefied *Z*-4FAzo dopant (Figure 1c(ii)). This liquid composite stores three forms of energy: the latent heat of eicosane, the latent heat of the liquid 4FAzo dopant, and the isomerization energy of the *Z*-isomer. The former two energies derive from ambient heat and photothermally converted energy, while the latter originates from the photon energy of green light. It is surprising that the composite maintains its liquid state even when cooled below eicosane’s crystallization point, with all stored energy fully retained within the liquid phase (Figure 1c(iii)). This exceptional thermal storage capability stems from metastable *Z*-liquid dopants, which inhibit eicosane crystallization through steric repulsion and intermolecular interactions. These dopants require optical activation to overcome the energy barrier for *Z*-to-*E* reversion. Upon blue-light exposure, the dopants rapidly revert to the *E*-configuration. This triggers crystallization of the composite, releasing the stored latent heat and isomerization energy as thermal energy, and restoring the system to its initial state (Figure 1c(iv)).

### 2.2. Photoisomerization Performance

**Figure 2:**
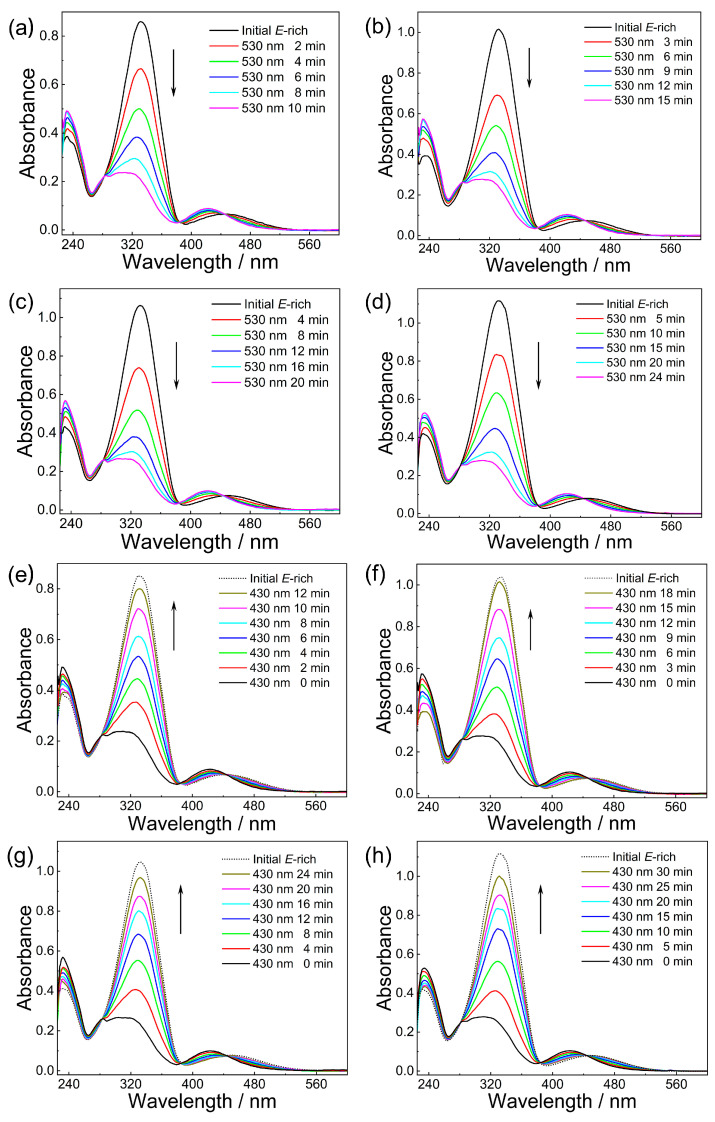
Time-evolved UV-Vis spectra of 4FAzo@eicosane composite under 530 nm (**a**–**d**) and 430 nm (**e**–**h**) light irradiation with different 4FAzo molar ratios: (**a**,**e**) 10%, (**b**,**f**) 20%, (**c**,**g**) 30%, and (**d**,**h**) 40%.

Investigating the photoisomerization behavior of 4FAzo@eicosane composites with different dopant ratios using UV–Vis absorption spectroscopy is essential, as irradiation under 530 nm and 430 nm light corresponds to the composite’s energy-harvesting (i.e., charging process) and heat-release (i.e., discharging process), respectively. As illustrated in Figure 2, all four composite samples exhibit identical initial UV-Vis spectra, featuring absorption peaks at 332 nm and 450 nm ascribed to π–π* and n–π* transitions, respectively. With increasing irradiation time under 530 nm light, the π–π* transition peak intensity notably decreases, whereas the n–π* transition peak slightly increases and exhibits a blue shift from 450 nm to 422 nm (Figure 2a–d). These spectral changes indicate *E*-to-*Z* isomerization of the 4FAzo dopant. Subsequent exposure to 430 nm irradiation triggers *Z*-to-*E* reversion, manifested by increased absorption at 332 nm and diminished intensity at 450 nm (Figure 2e–h). The photoisomerization behavior across different dopant ratios aligns with reported data for pure 4FAzo [35,47,49], confirming that eicosane incorporation does not adversely affect the photoisomerization behavior of the 4FAzo dopant. Notably, both energy-harvesting and heat-release processes reach photo-stationary states (PSS) faster in the composites than in pure 4FAzo (requiring 40 min) [35]. This kinetic acceleration suggests that the molecular interactions between 4FAzo and eicosane facilitate the configurational transitions. The primary reason for this is the insertion of the flexible alkyl chain at the para-position of 4FAzo into the lamellar arrangement of eicosane, which weakens the intermolecular π–π* stacking between adjacent Azos, increasing the free volume and thus enabling easier isomerization [31].

**Figure 3:**
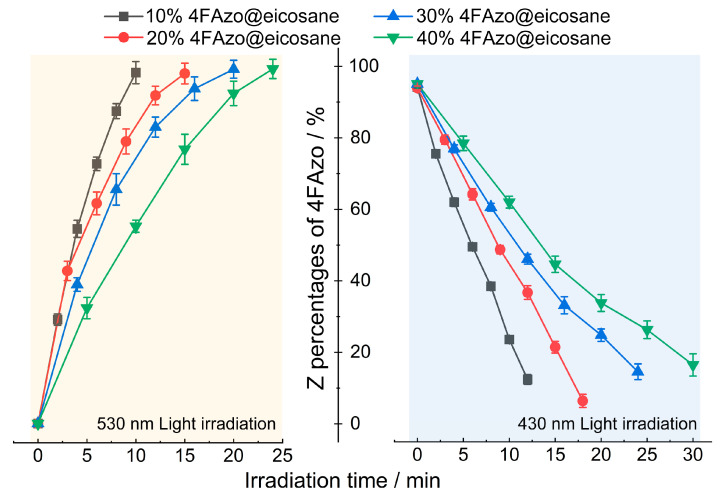
*Z*-isomer percentage of 4FAzo in 4FAzo@eicosane composites with different molar ratios vs. irradiation time under 530 nm (left) and 430 nm (right) light irradiation.

In order to further analyze the efficiency of energy harvesting and heat release processes, we monitored the evolution of the *Z*-isomer fraction under varying irradiation durations (the values were calculated using Equation S1). As shown in Figure 3 and Table 1, the 530 nm PSS achieves exceptional *Z*-isomer percentages of 97–99%, independent of 4FAzo molar ratio. That is competitive with the previously reported value (85%) [35], reconfirming eicosane’s facilitative role in 4FAzo isomerization. Notably, both energy-harvesting and heat-release processes exhibit prolonged PSS attainment times with increasing 4FAzo doping ratios. That attributed to the higher 4FAzo doping molar ratio would reduce the corresponding eicosane’s content, which intensifies the π–π stacking between adjacent 4FAzo molecules [15]. Consequently, the free volume provided by the phase-change material diminishes, and the longer alkyl chains become entangled, augmenting steric hindrance between molecules [56]. Ultimately, these factors conspire to impede the isomerization process within the 4FAzo@eicosane composite. This demonstrated that both free volume and steric hindrance significantly influence the isomerization rate.

### 2.3. Phase Transition Property

Using differential scanning calorimetry (DSC), we investigated melting and crystallization points of 4FAzo@eicosane composites with different doping molar ratios before and after green light (530 nm) irradiation and listed them in Table 2. As observed in Figure S4 and Table 2, all four groups of *E*-rich 4FAzo@eicosane composites exhibited similar phase transition behavior with melting points fluctuating within a small temperature range of 31 °C, below those of pure eicosane and pure *E*-rich 4FAzo dopant, which is a typical eutectic phenomenon. During solidification, two distinct exothermic crystallization peaks appear: the higher-temperature peak (approximately 24 °C) corresponds to eicosane’s liquid–solid transition [57,58], while the lower-temperature one represents crystallization of *E*-rich 4FAzo. Notably, as the 4FAzo dopant molar ratio increased and the crystallization peak position of eicosane remained relatively unchanged, whereas that of *E*-rich 4FAzo gradually moved to higher temperatures. This shift is attributed to the solvation effect of eicosane on the 4FAzo dopant: the higher the proportion of phase change material in the composites, the greater the degree of decrease in the crystallization point of dopants. After green light irradiation, the 4FAzo@eicosane composites displayed melting/crystallization properties analogous to pure eicosane (Figure S4, left column) since the photoinduced solid-to-liquid transition of 4FAzo dopant enables the *Z* isomer to maintain the liquid phase across a broad temperature range of −50 to 50 °C (Figure S4, Pure *Z*-rich 4FAzo).

**Figure 4:**
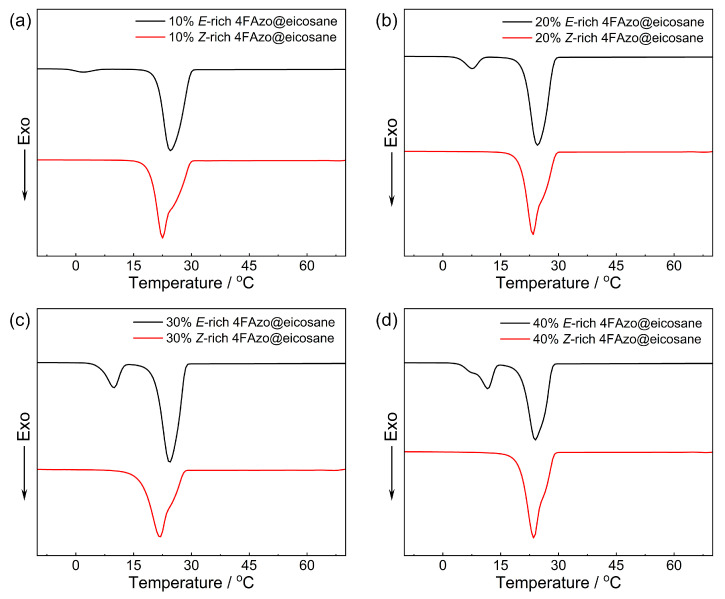
The DSC cooling curves of 4FAzo@eicosane composites before (*E*-rich) and after (*Z*-rich) green light irradiation at different dopant molar ratios: (**a**) 10%, (**b**) 20%, (**c**) 30%, and (**d**) 40% (cooling rate: 10 °C/min).

**Figure 5:**
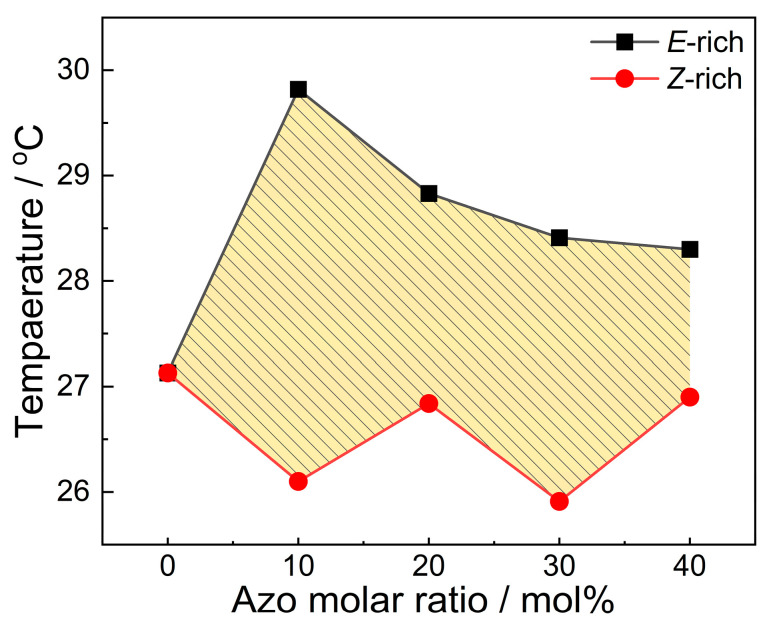
The crystallization temperature vs. dopant molar ratio for 4FAzo@eicosane composites in *E*-rich (before irradiation) and *Z*-rich (after green light irradiation) state. The shaded region indicates the supercooling temperature window enabling thermal energy storage and optically triggered heat release.

Given the critical importance of crystallization temperature for practical applications of phase change materials, we focused on crystallization temperature variations in 4FAzo@eicosane composites with different doping molar ratios before and after the charging process. As illustrated in Figure 4, all charged composites exhibited reduced crystallization temperatures compared to their uncharged counterparts. This reduction stems from the photoinduced *E*-crystal to *Z*-liquid transition of 4FAzo dopant, which enhances the intermolecular interactions between eicosane and dopant, while disrupting the originally ordered structure, thereby inducing supercooling [31,59]. Consequently, the 4FAzo@eicosane composite can maintain its liquid phase at temperatures below the eicosane’s crystallization point, enabling thermal energy storage under lower temperatures. Interestingly, the maximum supercooling was obtained at 10 mol% doping level, demonstrating that the supercooling degree can be tuned by adjusting the dopant molar ratio to meet diverse application requirements. The shaded area in Figure 5 establishes a supercooling temperature range for energy storage. The introduction of supercooling, achieved by the green light charging process, allows for thermal energy storage at lower temperatures. While subsequent blue-light irradiation triggers *Z*-to-*E* reversion to eliminate supercooling and enable optically controlled heat release.

### 2.4. Energy Storage Density

Energy density constitutes another critical performance metric for phase change materials. In 4FAzo@eicosane composites, the total energy density encompasses both phase-transition latent heat and isomerization enthalpy, which can be expressed using Equation (1) [15,34].

(1) $${∆H}_{total}=x{∆H}_{PCM}+\left(1-x\right){∆H}_{E-4FAzo}+\left(1-x\right){∆H}_{iso}$$

where ${∆H}_{total}$ represents the totally thermal energy released during the liquid to solid transition of 4FAzo@eicosane composite. ${∆H}_{PCM}$ and ${∆H}_{E-4FAzo}$ represent the latent heat of the eicosane- and *E*-rich 4FAzo, respectively; ${∆H}_{iso}$ represents the isomerization enthalpy of 4FAzo dopant from *Z*-to-*E* isomerization; and x is the content of eicosane in the 4FAzo@eicosane composite.

(1) Isomerization Enthalpy

**Figure 6:**
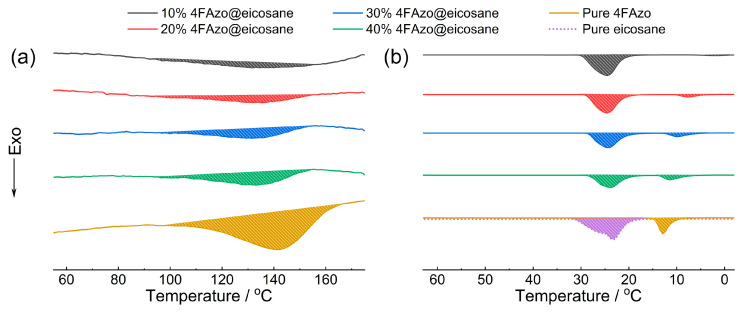
(**a**) The DSC exothermic curves of pure *Z*-rich 4FAzo molecule and *Z*-rich 4FAzo@eicosane composites with different dopant molar ratios from 60 to 180 °C (heating rate: 10 °C/min); (**b**) The DSC exothermic curves of liquid pure *E*-rich 4FAzo molecule, pure eicosane, and *E*-rich 4FAzo@eicosane composites with different dopant molar ratios from 180 to 0 °C (cooling rate: 10 °C/min).

The isomerization enthalpy of azobenzene is typically measured through thermally induced *Z*-to-*E* isomerization [60,61]. During the first heating stage for charged 4FAzo@eicosane composite, a broad exothermic peak appears in the temperature range from 90 to 150 °C, which is absent in the second heating cycle. This indicates that the exothermic peak corresponds to the *Z*-to-*E* isomerization energy of 4FAzo molecules, originating from the green light photon energy absorbed during the charging process. It can be observed from Figure 6a that the isomerization enthalpy increases proportionally with 4FAzo molar ratio in the composite, reaching 40.44 kJ/mol at 40% molar doping level. Compared to reported Azo-based photothermal energy storage materials, this isomerization enthalpy is not exceptionally high [26,62]. That may be because the alkyl chain at the para-position of 4FAzo molecule weakens intermolecular interactions, and the *Z*-isomer transforms into a liquid state upon charging, resulting in increased spatial volume and less compact molecular stacking compared to azobenzene materials with multiple hydrogen bonds [63,64].

(2) Latent Heat of Phase Change

This component comprises energy stored in both the liquid *E*-rich 4FAzo dopant and eicosane (Figure 6b). During the first cooling stage of the composite, the exothermic peak near room temperature corresponds to the eicosane crystallization energy, deriving from photothermal conversion of green light irradiation. The lower-temperature exothermic peak is associated with the crystallization of *E*-rich 4FAzo after the initial *Z*-to-*E* isomerization, with this energy originating from the *Z*-liquid passively absorbing ambient heat energy. The latent heat energy densities of the four composite samples, along with those of pure eicosane and 4FAzo dopant, are listed in Table S1. It is observed that as the 4FAzo doping ratio increases, the total latent heat energy density decreases. This is because the latent heat of pure 4FAzo is lower than that of eicosane. Consequently, as the doping level of 4FAzo rises, the relative content of eicosane decreases, leading to a reduction in the total latent heat energy density.

**Figure 7:**
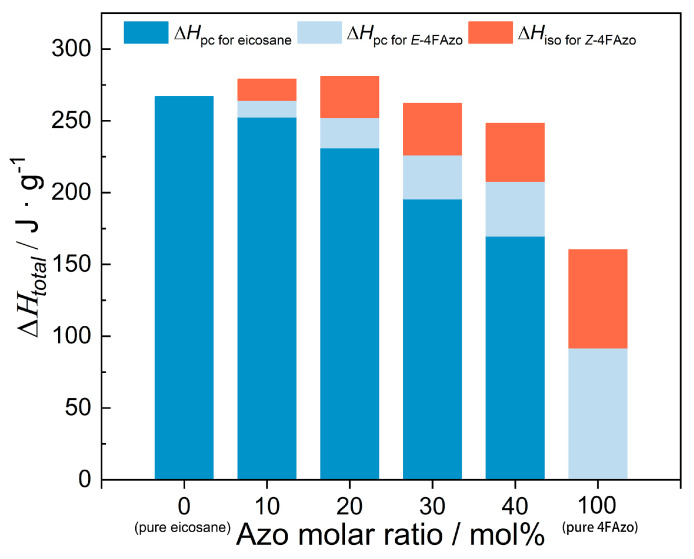
The total amount of released heat energy (Δ*H*_total_) for pure eicosane, 4Fazo, and 4FAzo@eicosane composites with different dopant molar ratios.

In order to provide a more intuitive understanding of the energy density trends in the 4FAzo@eicosane composites, the isomerization enthalpy and latent heat were merged, as depicted in Figure 7 and Table S1. Notably, the ${∆H}_{total}$ reaches a maximum of 280.76 J/g at a 20% molar doping ratio, surpassing that of pure eicosane [44,58]. This enhancement in energy density derives from synergistic molecular interactions between 4FAzo and eicosane and supplementary photochemical energy storage provided by the 4FAzo. This establishes a foundation for practical solar photothermal energy applications.

## 3. Materials and Methods

### 3.1. Materials

In this study, 2,6-difluoroaniline (99%), 3,5-difluorophenol (99%), sodium nitrite (NaNO_2_, 99%), sodium hydroxide (NaOH, 98%), 1-bromooctane (99%), potassium carbonate (K_2_CO_3_, 99.9%), and potassium iodide (KI, 99.8%) were purchased from Aladdin Biochemical Technology Co., Ltd. (Shanghai, China). Hydrochloric acid (HCl, AR), acetonitrile (ACN, AR), petroleum ether (PE, AR), ethyl acetate (EA, AR), and dichloromethane (DCM, AR and SP) were purchased from Titan Scientific Co., Ltd. (Shanghai, China). All the reagents were used directly without further purification.

### 3.2. Synthesis

The Azo molecule (*E*)-1-(2,6-difluoro-4-(octyloxy)phenyl)-2-(2,6-difluorophenyl)diazene (4FAzo) was synthesized via the azo coupling between substituted phenols and anilines, followed by Williamson ether synthesis. The detailed synthesis procedures and the characterization data included ^1^H and ^13^C NMR spectra (500 MHz INOVA spectrometer, Varian, Palo Alto, CA, USA), and Fourier transform infrared (FT-IR) spectrometry (Bruker Vertex 70, Bruker, Ettlingen, Germany) of the products are included in the Supplementary Materials (Figures S1–S3).

### 3.3. Preparation of 4FAzo@eicosane Composite Materials

The 4FAzo dopant and eicosane phase change material were mixed at predetermined molar ratios and dissolved in 5 mL of dichloromethane. The mixture was stirred continuously for 4 h at room temperature under dark conditions to ensure complete dissolution and homogeneous dispersion while preventing the photoisomerization of the 4FAzo molecules. Subsequently, the solution was dried at 35 °C for 12 h to allow complete evaporation of the solvent. This eventually yielded a series of 4FAzo@eicosane composite materials with different molar ratios.

### 3.4. UV–Vis Absorption Spectroscopy

The samples were irradiated using a 530 nm (20 Mw/cm^−2^) or 430 nm (60 Mw/cm^−2^) wavelength light source. The samples were positioned 5 cm below the light source, with the temperature set at 25 °C. During the irradiation, a small sample was taken with a glass rod (*Φ* = 0.5 mm) at regular intervals and the sample was dissolved in a cuvette filled with DCM solution, then the UV–Vis data were recorded, and the data obtained were normalized by using software 8.6.

### 3.5. DSC Measurements

The charged 4FAzo@eicosane composite was obtained by irradiation with 530 nm light for 30 min. The samples were subsequently transferred to a DSC pan to assess the phase change properties and exothermic characteristics. Unless noted differently in the figure descriptions, the DSC measurements were conducted with cooling and heating rates at 10 °C/min.

### 3.6. Statistical Analysis:

The data obtained in this study were used without preprocessing unless otherwise noted. The UV–Vis spectroscopy data are presented as mean ± standard deviation, with measurements from three independent experiments. All experiments were performed with multiple replicates (above triplicate).

## 4. Conclusions

A long-chain *tetra*-*ortho*-fluorinated Azo-doped eicosane composite was successfully prepared for visible light photothermal energy storage and optically-controlled heat release. The photoisomerization properties, optically-regulated phase transition performance, and energy storage densities of the 4FAzo@eicosane composite were studied in depth. The results demonstrated that doping 4FAzo into eicosane not only accelerated *E*-to-*Z* photoisomerization by reducing the intermolecular π–π* stacking between adjacent Azos to achieve 97–99% *Z*-isomer conversion but also modified eicosane’s crystallization behavior by introducing a photo-responsive energy barrier that induce supercooling (1.40–3.72 °C). Additionally, after green light charging, the supercooling degree and energy density can be tuned by adjusting the molar ratios of 4FAzo dopant in the composite. This allows photothermal energy storage at temperatures lower than the pristine crystallization point. Furthermore, under blue-light irradiation, supercooling is eliminated through *Z*-to-*E* isomerization, triggering the release of high-density (max 280.76 J/g) heat energy that included isomerization enthalpy of *Z*-to-*E* reversion, and phase change latent heat from eicosane and liquid *E*-4FAzo dopant, thus achieving the optically controlled heat release. Compared with pure eicosane and the reported Azo-based photothermal energy storage system, this energy release capacity is obviously higher. We further discussed the effects of variational molecular structures and molar ratios on the energy density and photoinduced supercooling and established a complete energy harvesting storage–release pathway for this system. This work creates new opportunities for near-room temperature solar thermal storage applications.

## Figures and Tables

**Table 1:** *Z*-isomer percentage of 4FAzo in composites with different molar ratios under various irradiation times of 530 and 430 nm.

4FAzo Ratios (mol%)	10		20		30		40	
530 nm	Irradiation Time (min)	*Z* Percent (%)	Irradiation Time (min)	*Z* Percent (%)	Irradiation Time (min)	*Z* Percent (%)	Irradiation Time (min)	*Z* Percent (%)
	2	29	3	43	4	39	5	32
	4	54	6	62	8	65	10	55
	6	73	9	79	12	83	15	77
	8	87	12	92	16	94	20	92
	10	98	15	97	20	99	24	99
430 nm	2	78	3	82	4	79	5	81
	4	63	6	65	8	61	10	63
	6	49	9	49	12	45	15	44
	8	37	12	35	16	32	20	32
	10	21	15	19	20	22	25	24
	12	9	18	2	24	11	30	13

**Table 2:** The melting, crystallization, and supercooling temperatures of 4FAzo@eicosane composites with different molar ratios before and after green light irradiation.

4FAzo Ratio (mol%)	Before 530 nm Irradiation (*E*-rich)			After 530 nm Irradiation (*Z*-rich)		Δ*T*_c_ (°C)
	*T*_m_ (°C)	*T*_c_ (°C)		*T*_m_ (°C)	*T*_c_ (°C)	
0 (pure eicosane)	32.86	27.13		32.86	27.13	
10	30.51	5.02	29.82	31.01	26.10	3.72
20	31.01	10.33	28.83	30.56	26.84	1.99
30	31.00	11.90	28.41	32.52	25.91	2.50
40	31.07	13.73	28.30	30.52	26.90	1.40
100 (Pure 4FAzo)	42.06	13.00		none	none	

*T*_m_ and *T*_c_ represent melting and crystallization point of sample, respectively. Δ*T*_c_ represents supercooling induced by *E*-to-*Z* isomerization.

## Data Availability

The original contributions presented in the study are included in the article, further inquiries can be directed to the corresponding author.

## Supplementary Materials

The following supporting information can be downloaded at: https://www.mdpi.com/article/10.3390/molecules30173576/s1, Figure S1: The ^1^H NMR spectrum of 4FAzo molecule; Figure S2: The ^13^C NMR spectrum of 4FAzo molecule; Figure S3: The FT-IR spectrum of 4FAzo molecule; Figure S4: The phase change behaviors of 4FAzo@eicosane composites with different molar ratios before (left) and after (right) charging process; Figure S5: The UV-Vis spectra of 4FAzo@eicosane composite under 365 nm light irradiation with different 4FAzo molar ratios: (a) 10%, (b) 20%, (c) 30%, and (d) 40%. Table S1: The total energy densities for 4FAzo@eicosane composites with 0%, 10%, 20%, 30%, 40%, and 100% doping molar ratios including latent heat of phase change for liquid eicosane and E-rich 4FAzo, and isomerization enthalpy of 4FAzo.

## Abbreviations

The following abbreviations are used in this manuscript:

OPCMs	Organic solid–liquid phase change materials
CPCMs	Composite phase change material
Azo	Azobenzene
4FAzo	(*E*)-1-(2,6-difluoro-4-(octyloxy)phenyl)-2-(2,6-difluorophenyl)diazene
DSC	Differential scanning calorimetry
