# Supplementary material for: Visible-Light-Controlled Thermal Energy Storage and Release: A Tetra-Ortho-Fluorinated Azobenzene-Doped Composite Phase Change Material
Source: Molecules. 2025 Aug 31;30(17):3576. doi: 10.3390/molecules30173576 (PMC12429928; doi:10.3390/molecules30173576)
Supplement: Supplementary file 1 [file molecules-30-03576-s001.zip › molecules-3803045-supplementary.pdf]

Supplementary Material

Visible-light-controlled thermal energy storage and release in tetra-ortho-fluorinated azobenzene-doped composite phase change materials

Yating Zhang [1,2], Jing Qi [1,3], Jun Xia [1], Fei Zhai [4,] and Liqi Dong [1,4,5,*]*

[1] Key Laboratory of Pollution Exposure and Health Intervention of Zhejiang Province, Interdisciplinary Research Academy, Zhejiang Shuren University, Hangzhou 310021, China

[2] College of Environment, Zhejiang University of Technology, 18 Chaowang RD, Hangzhou 310014, China

[3] Faculty of Social Sciences and Liberal Arts, UCSI University, Kuala Lumpur 56000, Malaysia

[4] Shandong Laboratory of Advanced Materials and Green Manufacturing at Yantai, Yantai Zhongke Research Institute of Advanced Materials and Green Chemical Engineering, Yantai 264006, China

[5] Zhejiang Collaborative Innovation Center for Full-Process Monitoring and Green Governance of Emerging Contaminants, Hangzhou 310021, China

Corresponding Author: zhaifei@amgm.ac.cn (F.Z.); liqi_dong@tju.edu.cn (L.D.)

Table of Contents

1. Characterization 2

2. Synthesis procedures 2

3. Structure characterization 3

4. Phase change behaviors 6

5. Table 7

6. Equation 8

1. Characterization

The SepaBean machine automated flash chromatography system (Santai Technologies, Inc.) was applied to purification. ^{1}H and ^{13}C NMR spectra were obtained on a 500 MHz INOVA spectrometer (Varian, USA) using dimethyl sulfoxide-d_6 or chloroform-*d* as the solvent and tetramethylsilane as the standard. IR spectra were obtained using Fourier transform (FT) IR spectroscopy and recorded using a Bruker Vertex 70 Fourier spectrometer with the powdered samples pressed into KBr pellets. The samples were charged/discharged under a controlled point LED lamp (HTLD-4II, Shenzhen Height-LED Opto-electronics Tech Co., Ltd.) with 530/430 nm wavelengths. The optical power meter (CEL-NP2000, Beijing China Education Au-light Co., Ltd.) was applied to measure the intensity of light. All UV-Vis absorption spectra were obtained using a UV-Vis spectrophotometer (V-750, JASCO, Japan). DSC measurements were conducted on a DSC 214 (Netzsch, Germany).

2. Synthesis procedures

(*E*)-4-((2,6-difluorophenyl)diazenyl)-3,5-difluorophenol (540 mg, 2 mmol, 1 eq), which was prepared as in our previous report [1], K_2CO_3 (553mg, 4 mmol, 2 eq), and KI (664mg, 4 mmol, 2 eq) were dissolved in 10 mL acetonitrile, and 1-bromooctane (0.417 mL, 2.4 mmol, 1.2 eq) was added dropwise. The solution was stirred at 90 °C for 2 hours. The reaction was monitored by TLC. Then, the reaction was stopped and the solution was cooled to room temperature, filtered, and concentrated on a rotary evaporator. The residue was purified with flash silica gel chromatography (hexanes: ethyl acetate = 30: 1). The final product was an orange solid (749 mg, 98%).

3. Structure characterization

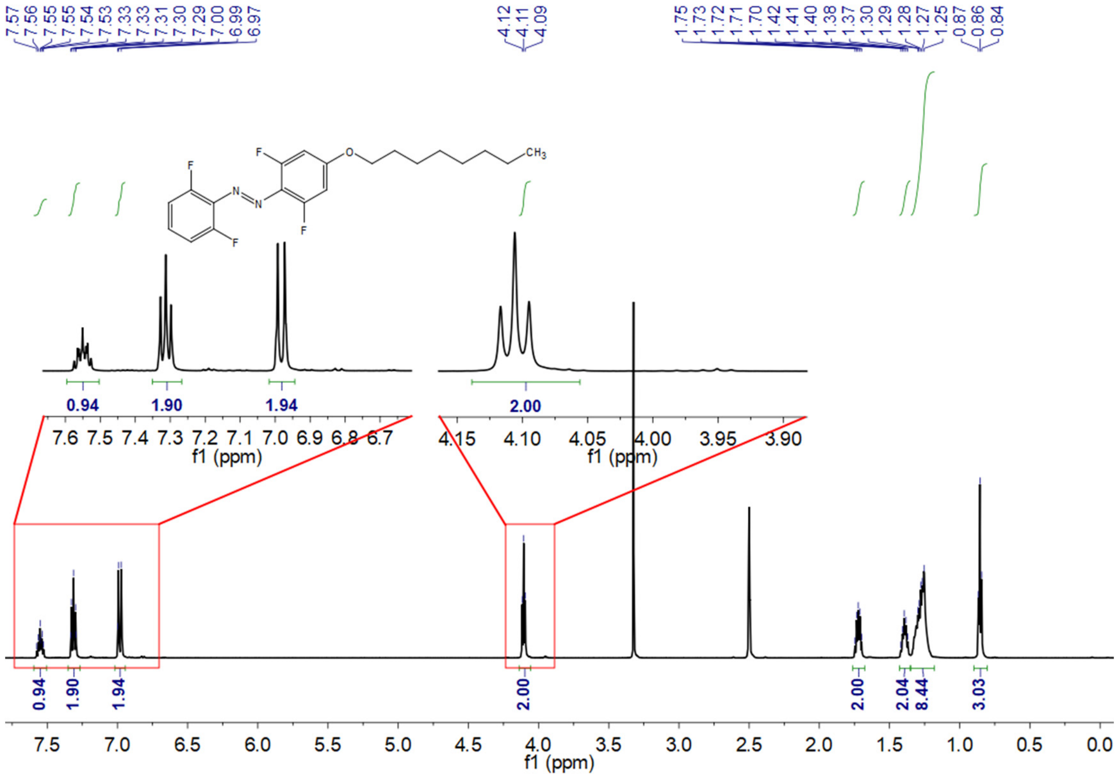

Figure S1. The ^{1}H NMR spectrum of 4FAzo molecule.

^{1}H NMR (400 MHz, DMSO-d_6): δ 7.60–7.50 (m, 1H), 7.31 (t, J = 8.9 Hz, 2H), 6.98 (d, J = 11.9 Hz, 2H), 4.11 (t, J = 6.6 Hz, 2H), 1.72 (p, J = 6.7 Hz, 2H), 1.39 (p, J = 7.0 Hz, 2H), 1.27 (dd, J = 14.3, 7.3 Hz, 8H), 0.90–0.80 (m, 3H).

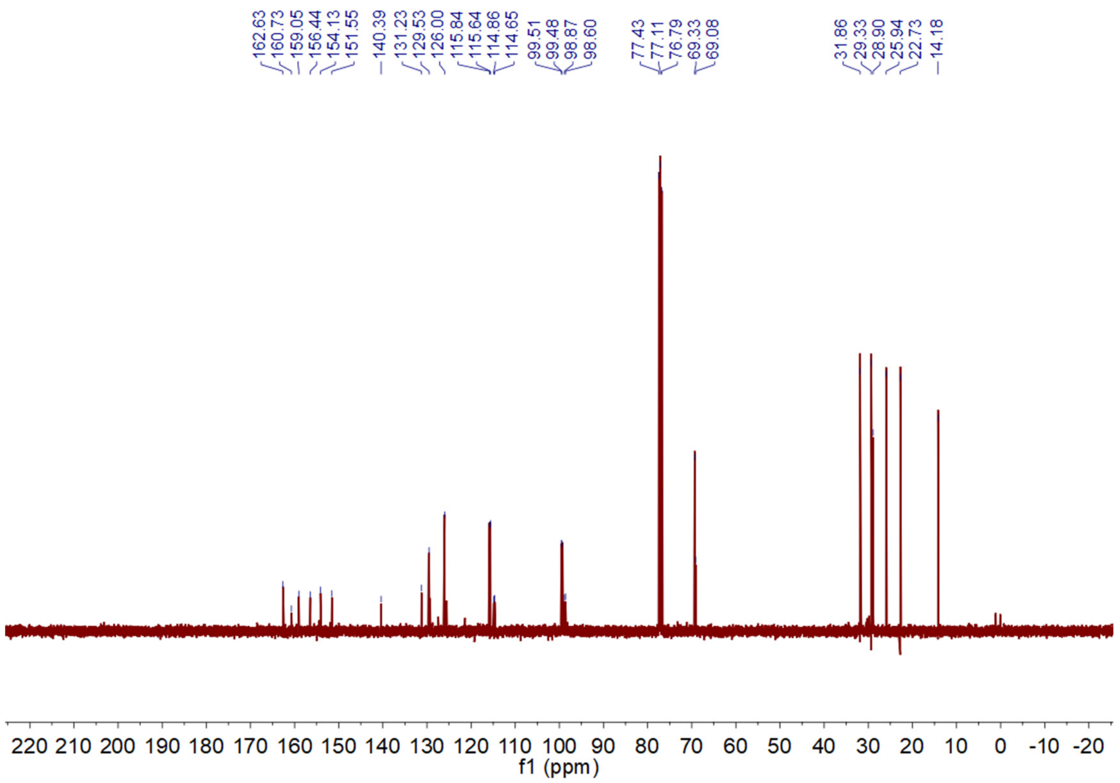

Figure S2. The ^{13}C NMR spectrum of 4FAzo molecule.

^{13}C NMR (101 MHz, Chloroform-*d*) δ: 162.63, 160.73, 159.05, 156.44, 154.13, 151.55, 140.39, 131.23, 129.53, 126.00, 115.84, 115.64, 114.86, 114.65, 99.51, 99.48, 98.87, 98.60, 77.43, 77.11, 76.79, 69.33, 69.08, 31.86, 29.33, 28.90, 25.94, 22.73, 14.18.

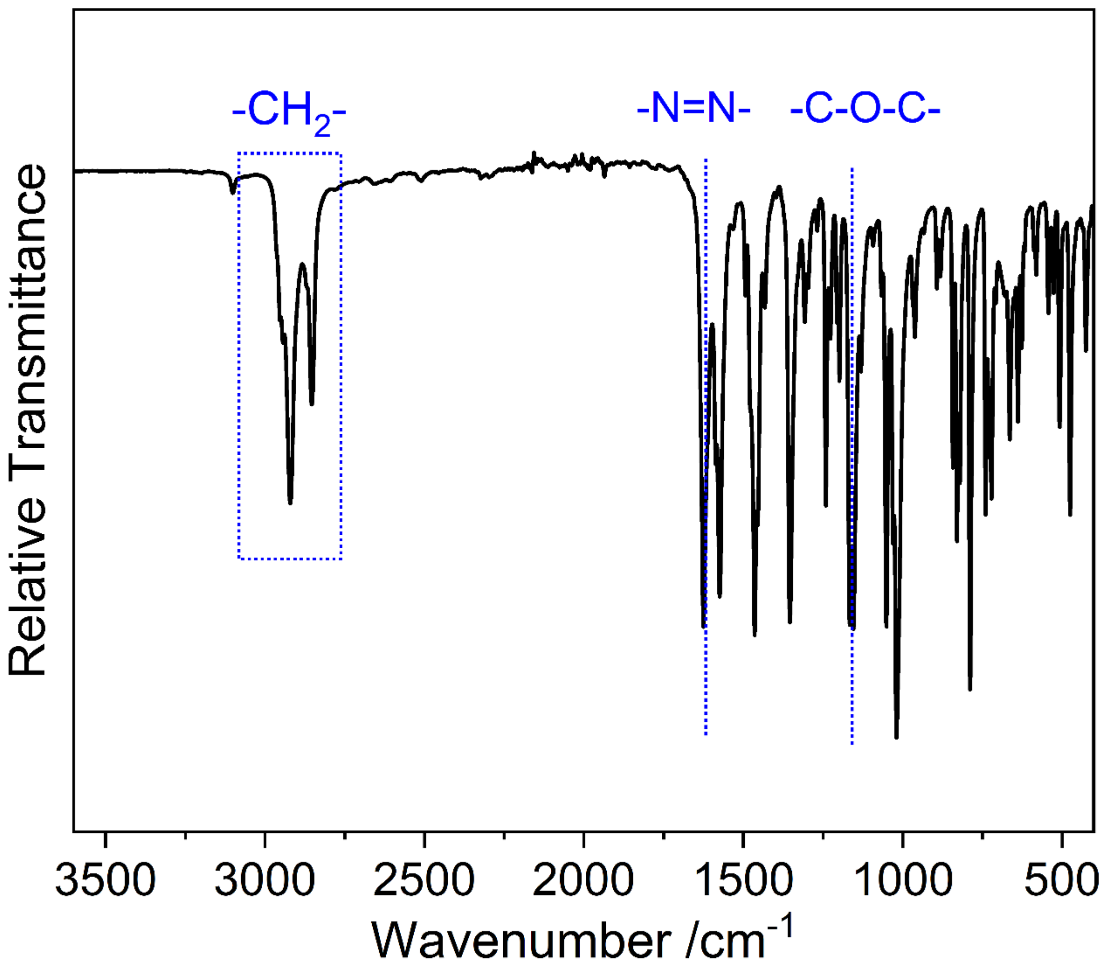

Figure S3. The FT-IR spectrum of 4FAzo molecule.

4. Phase change behaviors

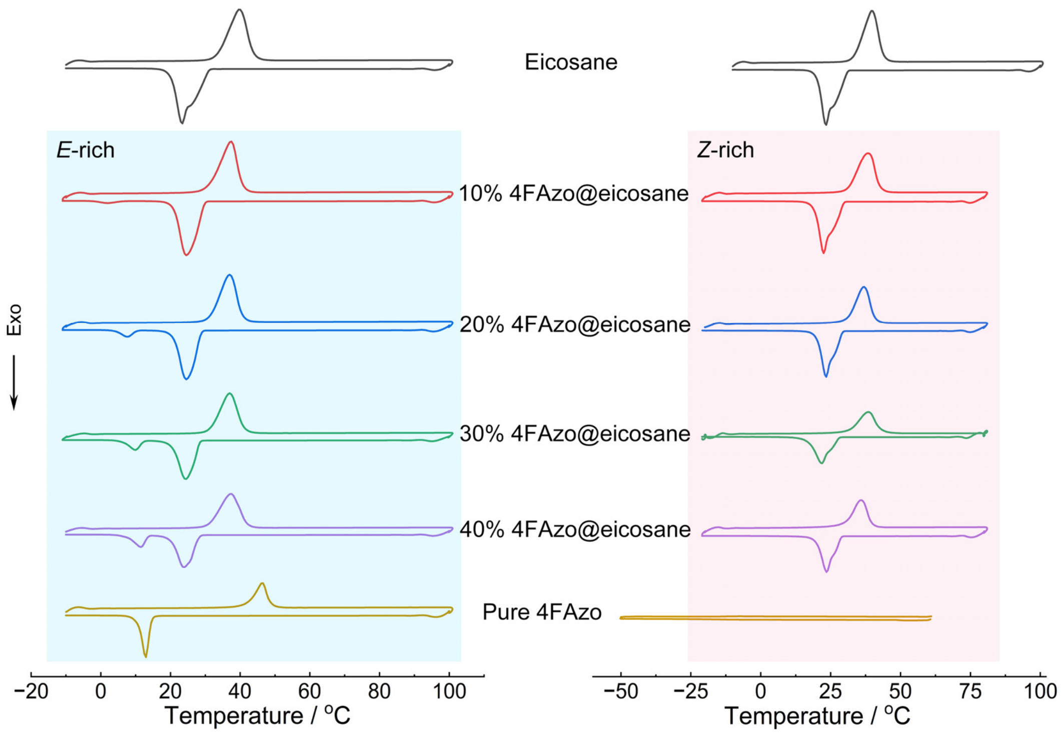

Figure S4. The phase change behaviors of 4FAzo@eicosane composites with different molar ratios before (left) and after (right) charging process.

5. Table

Table S1 The total energy densities for 4FAzo@eicosane composites with 0%, 10%, 20%, 30%, 40%, and 100% doping molar ratios, including latent heat of phase change for liquid eicosane and E-rich 4FAzo, and isomerization enthalpy of 4FAzo.

4FAzo ratio (mol%)	ΔH_{PC} (J/g)		ΔH_{iso} (J/g)	ΔH_{total} (J/g)
	ΔH_{PC} for eciosane	ΔH_{PC} for *E*-4FAzo	ΔH_{iso} for Z-4FAzo	
0 (pure eicosane)	266.80	0.00	0.00	266.80
10	252.60	11.68	14.61	278.89
20	231.10	21.16	28.50	280.76
30	195.70	30.63	35.67	262.00
40	169.8	38.05	40.44	248.29
100 (pure 4FAzo)	0.00	91.82	68.25	160.07

6. Equation

Equation S1. Percentage of Z-isomers

According to the UV-Vis spectra, the percentage of isomers can be estimated as the percentage change of absorbance at the wavelength of the π-π* transition peak, as follows:

$$Z\ (\%) = \frac{A_t - A_E}{A_Z - A_E} \times 100\% \quad \text{(S1)}$$

where A_t is the absorbance intensity of samples at the irradiation time of "t"; A_E is the absorbance intensity of 100% *E*-isomer. A_Z is the absorbance intensity of 100% *Z*-isomer. The initial state was assumed to be ~100% *E*-isomer, while the 365-nm PSS in dilute solutions was assumed to be ~100% *Z*-isomer.

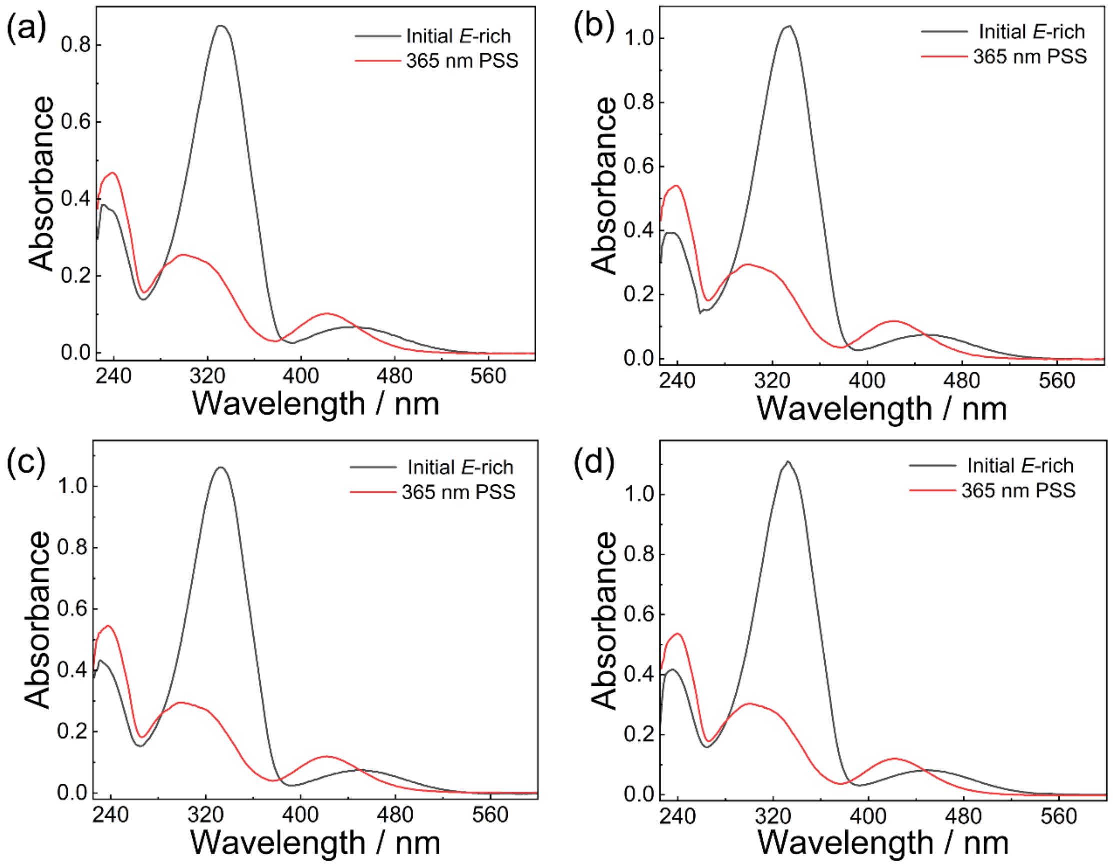

Figure S5.The UV-Vis spectra of 4FAzo@eicosane composite under 365 nm light irradiation with different 4FAzo molar ratios: (a) 10%, (b) 20%, (c) 30%, and (d) 40%.

Reference

1. Wu, Y.; Dong, L.; Tang, S.; Liu, X.; Han, Y.; Zhang, S.; Liu, K.; Feng, W. An innovative azobenzene-based photothermal fabric with excellent heat release performance for wearable thermal management device. Small 2024, 20, 2404310.
